# Where Can Patients Obtain Information on the Preapproval Access Pathway to Investigational Treatment in Japan? A Survey of Patient Advocacy Organizations’ Websites

**DOI:** 10.1002/cpdd.745

**Published:** 2019-10-08

**Authors:** Haruka Nakada, Kyoko Takashima

**Affiliations:** ^1^ Division of Bioethics and Healthcare Law Center for Public Health Sciences National Cancer Center Chuo‐ku Tokyo Japan; ^2^ Medical Genomics Center National Center for Global Health and Medicine Shinjuku‐ku Tokyo Japan

**Keywords:** clinical trials, expanded access, investigational drug, patient advocacy organization, preapproval access

## Abstract

Investigational treatments are those that have been approved for testing in humans but are not yet available as an approved treatment option. For many patients with a terminal illness who have no approved treatment option and are not eligible for a clinical trial, investigational treatments are the last resort. However, not much is known about the dissemination of information by patient advocacy organizations (PAOs). We evaluated the quantity and quality of information on preapproval access to investigational therapies provided by Japanese PAO websites between January 24 and March 29, 2019. A total of 49 PAOs were identified. Of these, 16 (33%) provided no relevant information. The most frequent information provided was the PAO's own clinical trial finder or list of clinical trials (n = 15, 31%); of the 10 cancer‐related PAOs, 5 (50%) provided this information. Nine (18%) PAOs had developed patient registries or provided a link to relevant registries. Only 1 PAO (2%) provided a link about the Ministry of Health, Labour, and Welfare trials that described the process and regulations of clinical trials. Our results indicate that PAOs do not disseminate adequate information on preapproval pathways. We suggest that the government involve PAOs in disseminating this information to both patients and physicians.

Preapproval access to investigational treatments is considered as a “last resort” for patients who have exhausted approved treatment options. The main way to gain preapproval access to investigational treatment is by participating in clinical trials. However, there are various barriers to trial participation, including access to a clinic, eligibility, physicians’ attitudes toward clinical research,^1^ the types of treatment centers,^2^ and patients’ age.^3^ By reducing these barriers, patients can have equal access to clinical trials, and investigators can conduct clinical trials more smoothly.

Patients who cannot participate in clinical trials often seek alternative ways to receive investigational treatment. The US Food and Drug Administration has developed an expanded access program that, since 1938, has allowed patients with a terminal illness to request access to investigational treatments.^4^ In addition, a federal right‐to‐try act was enacted in May 2018, which has provided terminally ill patients with a new pathway to receive investigational treatments.^5^ In the European Union, the European Medicines Agency provides recommendations for Member States to develop their own legal framework of compassionate use (CU).^6^ Compared with the United States and the European Union, in India, CU is in its nascent stage owing to the lack of policies and laws to govern it,^7^ and China has been developing a CU pathway.^8^ The Ministry of Health, Labour, and Welfare (MHLW) in Japan has introduced 2 preapproval pathways to investigational treatments in 2016, including the expanded trial (ET)^9^ and the patient‐requested therapy system (PRTS).^10^ Both are conducted within a framework of clinical trials. The ET is a Japanese CU program that allows patients who have exhausted approved therapies to receive an investigational one that is currently under development in a clinical trial, even though they are not eligible for the trial. The PRTS is also a CU‐like program that provides patients with quicker access to an investigational treatment via a patient's request to a designated hospital. The designated hospital develops a clinical trial protocol based on the request and submits it to the MHLW committee. The patient can receive the investigational treatment by participating in the clinical trial that the designated hospital has developed after the committee's approval.

The PRTS can be beneficial for patients who have exhausted approved treatment options and eligible clinical trials inside Japan, such as patients with cancer.^11^ Since the PRTS was introduced, there have been 112 inquiries about investigational treatment from patients to designated hospitals; however, only 7 investigational treatments have been approved by the MHLW committee, which is less than the government had expected. The PRTS has been underutilized, even though patients can expand treatment options by themselves.

Patients’ low awareness of the PRTS could be one of the possible challenges of using it. Patients and families may go to patient advocacy organization (PAO) websites to seek information about their conditions and possible treatment options; however, little is known about the PAO's role as an information source on preapproval access to investigational therapies. To our knowledge, there has been no report analyzing how the Japanese PAO website provides information on how to receive investigational treatments. In this study, we analyzed Japanese PAO websites to evaluate the quantity and quality of information on preapproval access to investigational therapies.

## Methods

As we collected the data from PAO websites, which are open to the public, an ethics review was not required by the Ethical Guidelines for Medical and Health Research Involving Human Subjects in Japan.

We selected PAOs that are members of the Japan Patients Association, National Association of Cancer Patient Groups, or Genetic Alliance JP, or that are listed on the Japan Intractable Diseases Information Center website or the Community for Patient Participation in Japan. Organizations were excluded if they were not disease specific, did not have websites, contained the name of a person or patient, used regional areas in the name (eg, “Cancer Patients and Family Association in Tokyo”), or were regional branches of an organization. Ultimately, 49 PAOs were identified as eligible subjects, and we confirmed that they were classified as nonprofit organizations.

We reviewed whether the 49 PAO websites contained the following information on preapproval access pathways to investigational drugs: links to clinical trial databases; the PAO's own clinical trial finder or list of clinical trials; the PAO's patient registry for future research; lists of investigational drugs available for a preapproval access pathway, including clinical trials, the ET, or the PRTS; and information on the PRTS and/or ET, position/commentary on the preapproval access pathway, and the MHLW or Pharmaceuticals and Medical Devices Agency (PMDA) resources regarding the preapproval access pathway to investigational medical products. Data collection occurred from January 24 to March 29, 2019.

## Results

The disease areas of the 49 PAOs included 10 cancers (n = 10, cancer‐related PAOs), and 37 other diseases (n = 39), most of which were rare diseases. The PAOs’ website contents pertaining to preapproval access are shown in Table 1. Among the PAOs, 16 (33%) did not provide any relevant information on preapproval access to investigational medical products.

**Table 1:** Contents of the PAOs’ Websites

	Yes		No		Others	
Information Category	n	(%)	n	(%)	n	(%)
Link to clinical trial database	4	(8)	45	(92)	0	(0)
	1	(10)	9	(90)	0	(0)
PAO's own clinical trial finder or list of clinical trials	15	(31)	33	(67)	1^a^	(2)
	5	(50)	5	(50)	0	(0)
PAO's patient registry rather than trial finder	9	(18)	36	(73)	4^b^	(8)
	2	(20)	8	(80)	0	(0)
Lists of investigational drugs for clinical trials or preapproval access pathway	9	(18)	40	(82)	0	(0)
	2	(20)	8	(80)	0	(0)
Information on patient‐requested therapy system, expanded trial	2	(4)	46	(94)	1^c^	(2)
	1	(10)	9	(90)	0	(0)
Position/Commentary on preapproval access pathway	4	(8)	45	(92)	0	(0)
	1	(10)	9	(90)	0	(0)
MHLW or PMDA resources provided	1	(2)	41	(84)	7^d^	(14)
	0	(0)	10	(100)	0	(0)

MHLW, Ministry of Health, Labour and Welfare; PAO, patient advocacy organization; PMDA, Pharmaceuticals and Medical Devices Agency

The number of PAOs’ websites in general is in the upper row (N = 49); that of PAOs’ websites in cancer area is in the lower row (n = 10).

a One PAO provided a broken link in a tab showing “a list of clinical trials.”

b PAOs recruited participants to clinical research other than clinical trials, such as biobanking.

c One PAO provided information on a specific committee at MHLW to call for requests for research and development of unapproved drugs in Japan.

d PAOs had links to the home page of MHLW, PMDA, or the page of a specific committee, not the specific page providing information on preapproval access pathways.

The most frequent information provided was “PAO's own clinical trial finder or list of clinical trials” (n = 15; 31%). The PAOs provided information regarding clinical trials relevant to their diseases from pharmaceutical company press releases, research institutions, and personal communication with researchers. Nine PAOs (18%) had developed their own patient registries or provided a link to registries targeting their relevant diseases to collect potential clinical trial participants.

Some PAOs followed the process of introducing preapproval access, especially the PRTS. Four PAOs (8%) pointed out the potential negative impacts on the universal health care system when introducing the PRTS. In particular, these PAOs were concerned that the PRTS might cause a delay in regulatory approval. Patient's medical cost is partially reimbursed by the national health insurance if the treatment has been approved by the PMDA. Introducing the PRTS might also cause inequality in patients’ access to investigational therapies because of their economic circumstances. Another concern was protection of patient safety, as introduction of the PRTS could increase the number of patients who would be exposed to investigational therapies with unproven safety and efficacy.

Only 1 PAO (2%) provided a link to an information source about MHLW clinical trials that described the process and regulations of clinical trials, informed consent, and instructions for potential participants. No PAO provided a link to the MHLW or PMDA website that included information on preapproval access pathways.

Cancer‐related PAOs (n = 10) provided similar information to the overall trend, except for 1 category, “PAO's own clinical trial finder or list of clinical trials.” Five PAOs (50%), which represented a higher percentage than the overall trend, provided their own list of relevant clinical trials. In addition, 1 of the cancer‐related PAOs offered detailed information on clinical trials, including both clinical trial databases and its own list of clinical trials with instructions for conducting effective searches, including keywords and a search example. Another cancer‐related PAO provided information on the preapproval access pathway via ways other than clinical trials, and pointed out the negative impact by the PRTS on the universal health care system; this is the only PAO that provided descriptions of the ET and PRTS.

## Discussion

Our study showed the current situation of how information on preapproval access to investigational treatments—such as clinical trials, the ET, and the PRTS—are disseminated by Japanese PAO websites. The PAOs in our study rarely provided the relevant information on preapproval access to investigational treatments on their websites. These results indicate that information regarding the preapproval access pathway has not spread among PAOs in Japan, even though 3 years have passed since the new preapproval access pathways were introduced.

The initial method of accessing preapproval medical products is through clinical trial participation; however, >90% of the PAOs in our study did not provide a link to any clinical trial database. This could be because of the complex structure and contents of clinical trial databases in Japan; there are 4 major and/or national clinical trial databases. Patients may find it difficult to decide which database to use and to search for clinical trials that are relevant to their diseases because these databases do not have instructions for users who are not medical experts. Only 4 PAOs in our study provided a link to a clinical trial database, and 1 PAO provided directions to conduct an effective database search (eg, specific keywords, examples of clinical trial searches). Thus, it appears that major or national clinical trial databases are limited information sources for patients.

Compared with the major clinical trial databases, a clinical trials finder or lists of clinical trials focusing on specific diseases would be useful for patients and their families; we found that PAOs more often had their own clinical finder or lists rather than links to the major clinical trial databases. These resources would make it easier to locate disease‐specific clinical trials than searching the clinical trial databases. PAOs that provide the finder or lists are limited because maintaining updated information imposes a huge burden in terms of cost, medical knowledge, and human resources. Some PAOs in our study, especially those that target rare diseases, have provided research information regarding not only clinical trials but also biobanking and other clinical research by collaborating with researchers. Because there are many active cancer clinical trials, cancer‐related PAOs can collect more information on clinical trials compared with rare diseases.

Other preapproval access pathways in Japan are the PRTS and ET; however, the studied PAOs did not provide much information about these pathways. Thus, it seems that PAOs pay little attention to new preapproval access pathways, even though patients can request to use investigational therapies themselves through the PRTS. There are 2 possible reasons for the PAOs’ lack of information concerning this.

First, concerns about preapproval pathways—especially the PRTS—might outweigh the potential benefits for patients. Before introducing the PRTS, the PAOs we investigated expressed concern that it would violate the universal health care system. If many patients use the PRTS to receive an investigational treatment, the regulatory approval might be delayed, and the medical cost of the treatment might remain unreimbursed by the national insurance until it is approved by the PMDA; this situation could increase the patients’ financial burden. Once the investigational treatment has been approved and covered by the national health insurance, the patients’ financial burden would be decreased because part of the medical cost would be reimbursed. Therefore, patients require prompt regulatory approvals and insurance coverage rather than the expansion of preapproved access pathways.

Second, the information sources for preapproval pathways are limited. For example, during our data collection period, the MHLW had developed a rudimentary website for the PRTS, but the information provided was intended for a mix of both patients and medical experts. In April 2019, the MHLW created separate websites for patients and for medical experts. The media, especially the mass media, have rarely reported on the PRTS. As a result, patients, families, and even medical staff do not have sufficient information sources about preapproval access pathways. A few of the PAOs in our study provided information on preapproval pathways; however, they provided a background of the PRTS rather than a description of the system itself. In summary, our findings show that the MHLW's websites have not been sufficiently used by PAOs as an information source of preapproval access to investigational medical products. The information delivery strategy in this area therefore needs improvement.

Preapproval access pathways can provide posttrial access to investigational treatments to the patients. Even after the completion of a clinical trial, it takes time to review the clinical trial data before a regulatory authority makes a new drug available on the market; however, patients with a severe condition who have experienced a significant health benefit from a trial drug do not have the luxury of time. In addition, after trial completion in low‐ or middle‐income countries, participants often do not have access to the drug because of the marketing strategies of pharmaceutical companies—the drug might remain unapproved in these countries while on the market in other countries in which the pharmaceutical companies can expect larger financial sales of the drug. One possible solution would be to develop a posttrial policy for clinical trials.^12^ In India, there have been some cases that caused an access gap to the investigational drug availabilities between developing and developed countries after the completion of the clinical trials. The policy, including ethics guidelines, can clarify the sponsors’ responsibility of providing preapproval access after completion of clinical trials. Another solution would be to develop a specific regulation for the preapproval access pathway that includes CU.^7^ In the era of personalized medicine, the need for both posttrial access and preapproval access pathways is increasing.^13^ As there has been a growing trend of international collaboration of drug development, posttrial and preapproval access to the investigational drug would become an important issue for clinical trials’ sponsors and patients participating in clinical trials.

PAOs’ role as an information source is increasing in the Internet era. The quantity and quality of information on clinical trials, the ET, and the PRTS need to be improved so that patients can make more informed decisions about possible treatment options.

## Conclusions

Despite the time lag in anticancer drug approval in both Japan and the United States for rare cancers,^14^ we found that cancer‐related PAOs do not disseminate information on preapproval access to investigational drugs in Japan because they do not provide enough information sources. As cancer is one of the leading causes of death in Japan,^15^ the need for tumor‐profiling multiplex gene panel tests for providing possible treatment options, including investigational treatments, will increase. Indeed, patients with cancer and their families have been reported to recognize the benefits of these tests,^16^ and the need for preapproval access to investigational anticancer drugs will also increase. In addition to governmental resources, PAOs are expected to be valuable sources of information on preapproval pathways for patients and families.

Our study has 3 limitations: First, we reviewed the contents of only publicly available websites. PAOs may share more information on members‐only pages, which we were unable to access. Second, we are unaware of PAOs’ specific policies for their website contents. PAOs may avoid providing preapproval pathway information based on these policies. For example, if an executive board of a PAO decides to oppose preapproval access, they may avoid providing relevant information on their website. Third, some PAOs may focus more on peer support rather than on sharing policy or regulatory information. Future study could investigate PAOs’ attitudes toward preapproval access.

Based on our results, we suggest that the government involves PAOs in disseminating information on preapproval pathways. For example, governments could provide information tool kits, such as a website to help both patients and physicians navigate information on how to use the preapproval access program^17^ or a documentation‐supporting system for physicians. In addition, the government should share information on the performance of preapproval access pathways with patients and physicians. PAOs would then be able to evaluate whether the pathways would be useful for the patients in their disease area based on actual cases.

## Conflicts of Interest

The authors declare that they have no conflicts of interest to disclose.

## Funding

This work was supported by JSPS KAKENHI Grant Number 17K17665 and 15H05913.

## Data Sharing

The data for this study are available from the authors by email; however, the data are in Japanese.
